# Letter-to-the-editor on “A low-carbohydrate diet induces hepatic insulin resistance and metabolic associated fatty liver disease in mice”

**DOI:** 10.1016/j.molmet.2024.101898

**Published:** 2024-02-13

**Authors:** Xin Wang, Bo Jiang

**Affiliations:** Beijing Tiantan Hospital, Capital Medical University, China; Beijing Shijitan Hospital, Capital Medical University, China; Beijing Tiantan Hospital, Capital Medical University, China

Dear editors of Molecular Metabolism,

We read with great interest the article by Wolfrum C, Challa TD et al., concerning “A low-carbohydrate diet induces hepatic insulin resistance and metabolic associated fatty liver disease in mice” [1]. We believe, however, that certain aspects require further consideration.

Firstly, the author asserted that that ketogenic diet (KD), as compared to high-fat diet (HFD), causes liver fibrosis, hepatic insulin resistance and nonalcoholic steatohepatitis (NASH), regardless of body weight loss, through a possible new mechanism involving IL-6-JNK signaling at thermoneutrality. They posited a novel mouse model for NASH, treated with KD at thermoneutrality, which seemed to contradict established logic. This conclusion might not account for the known proinflammatory responses at thermoneutrality, regardless of whether the body was in ketosis or not, there was an increase in macrophage infiltration and the corresponding pro-inflammatory cytokines (IFN-γ, TNF-α, IL-1β, and IL-6) in the brown adipose tissue of mice [2,3]. Thus, the study might overgeneralize the negative impacts of KD, potentially introducing bias into its findings.

Secondly, the premise of KD being detrimental without considering total energy intake is flawed. The ketogenic diet is characterized by a low carbohydrate content (<50 g/day),15–30 g of fat/day, but the total daily energy intake varies according to different records. There is a type of ketogenic diet that reduces overall intake, called the very-low-calorie ketogenic diet (VLCKD), which involves controlling total calories to less than 800 kcal/d [4,5]. While others maintain that the energy intake on a KD typically exceeds 1000 kcal/day [6]. Others find that on KD, a calorie restriction below 1200 kcal/day on KD can significantly reduce body weight and hepatic steatosis improvement [7]. In summary, the different total calories intakes from KD lead to entirely different prognoses. Most data for a significant remission in non-alcoholic fatty liver disease (NAFLD) came from a combination of KD and calorie restriction (hypocaloric diet) [7]. The beneficial effects of KD without a low-calorie diet remain to be determined. Why not use an energy-restricted KD for experiments in this work? Certainly, the debate focuses on whether the reduction in hepatic lipid infiltration is caused by the KD pattern or general weight loss [8], or whether the restriction of carbohydrate intake leads to relatively lower blood glucose levels, thereby reducing insulin levels and in turn decreasing de novo lipogenesis in the liver [9]. In this regard, Mardinoglu et al. [10] observed a significant reduction in hepatic fat content of 43.8% in 10 Nordic patients with NAFLD after KD intervention, without total energy restriction (3,115 kcal/day on average). Moreover, the authors contend that the protein content in KDs used clinically in humans is limited. In reality, in KD regimens, the quantity of high biological value proteins (from peas, milk, soy, and whey) is between 0.8 and 1.2 g per kilogram of ideal body weight, to maintain lean mass and meet the minimum daily physical requirements [11]. The notion that KD is a low-protein diet is baseless. Since in this study, the C57BL/6 mice used were all healthy, would mainstream nutritionists advocate for the application of a KD to the healthy populations. The authors do not claim that the total energy intake during the feeding period was the same between the two groups, which also reduces the credibility of the results. The limitations of study design should also be considered as a potential confounding factor. Particularly, their overly simplistic presentation of the underlying biology was reflected in using ketogenic mice as a new NASH mouse mode.

Thirdly, it was unfortunate that the authors failed to conduct a thorough review of the literature, which reveals numerous studies contradicting their viewpoints. A review entitled “Current Evidence Concerning Effects of Ketogenic Diet and Intermittent Fasting in Patients with Nonalcoholic Fatty Liver” [7] summarized the efficacy of these trendy dietary patterns in treating NAFLD concerning intrahepatic fat content, fibrosis, liver enzymes, and might offer us a different perspective in guiding treatment for NAFLD patients: ketogenic diets and intermittent fasting reduce body weight, mitigate the progression of hepatic steatosis, and improve liver fibrosis. Another study [11] revealed that KD has a significant impact on reducing weight, fat mass, waist circumference, total cholesterol, and triglyceridemia, as well as improving insulin resistance, compared to other diet interventions of the same duration. Moreover, based on several current evidence, KD indeed benefits patients with NAFLD and other metabolic issues [[12], [13], [14]].

In conclusion, the scientific rationales behind KD patterns, as well as safety concerns for certain patient populations, remain contentious. In this study, factors potentially leading to divergent outcomes that may not have been sufficiently emphasized include the types of fats and proteins consumed in the KD, as well as the insufficiency or deficiency of micronutrients related to low carbohydrate intake, which could elicit varying metabolic responses. Ketogenic diet are often high in saturated fats, potentially elevating low-density lipoprotein (LDL) cholesterol levels and insulin resistance, thereby possibly augmenting the risk of atherosclerotic cardiovascular diseases and type 2 diabetes [11]. Consequently, KD should be conducted under professional medical supervision with strict adherence to the protocol. It is imperative to frequently monitor health markers (lipids profile and blood glucose, especially) and to provide meticulous micronutrient supplementation, including B-complex vitamins, vitamin C and E, minerals (potassium, sodium, magnesium, and calcium) and omega-3 fatty acids, during the period of KD [15]. We believe that KD, as a nutritional and metabolic intervention, indeed represents a superior tool for the treatment of obesity and its related comorbidities. However, the use of KD demands further refinement and standardization.

## CRediT authorship contribution statement

**Xin Wang:** Investigation, Writing – original draft. **Bo Jiang:** Conceptualization, Writing – review & editing.

## Declaration of competing interest

The authors declare that they have no known competing financial interests or personal relationships that could have appeared to influence the work reported in this paper.

## Data Availability

No data was used for the research described in the article.
